# JI017 Attenuates Oxaliplatin-Induced Cold Allodynia via Spinal TRPV1 and Astrocytes Inhibition in Mice

**DOI:** 10.3390/ijms22168811

**Published:** 2021-08-16

**Authors:** Ji Hwan Lee, Hyunseung Ji, Seong-Gyu Ko, Woojin Kim

**Affiliations:** 1 Department of Physiology, College of Korean Medicine, Kyung Hee University, Seoul 02447, Korea; mibdna@khu.ac.kr; 2 Department of East-West Medicine, Graduate School, Kyung Hee University, Seoul 02447, Korea; ghost0929@naver.com; 3 Korean Medicine-Based Drug Repositioning Cancer Research Center, College of Korean Medicine, Kyung Hee University, Seoul 02447, Korea; epiko@khu.ac.kr

**Keywords:** allodynia, astrocyte, JI017, medicinal herbs, oxaliplatin, TRPV1

## Abstract

Oxaliplatin, a well-known chemotherapeutic agent, can induce severe neuropathic pain, which can seriously decrease the quality of life of patients. JI017 is an herb mixture composed of *Aconitum carmichaelii*, *Angelica gigas*, and *Zingiber officinale*. Its anti-tumor effect has been reported; however, the efficacy of JI017 against oxaliplatin-induced allodynia has never been explored. Single oxaliplatin injection [6 mg/kg, intraperitoneal, (i.p.)] induced both cold and mechanical allodynia, and oral administration of JI017 (500 mg/kg) alleviated cold but not mechanical allodynia in mice. Real-time polymerase chain reaction (PCR) analysis demonstrated that the upregulation of mRNA of spinal transient receptor potential vanilloid 1 (TRPV1) and astrocytes following oxaliplatin injection was downregulated after JI017 treatment. Moreover, TRPV1 expression and the activation of astrocytes were intensely increased in the superficial area of the spinal dorsal horn after oxaliplatin treatment, whereas JI017 suppressed both. The administration of TRPV1 antagonist [capsazepine, intrathecal (i.t.), 10 μg] attenuated the activation of astrocytes in the dorsal horn, demonstrating that the functions of spinal TRPV1 and astrocytes are closely related in oxaliplatin-induced neuropathic pain. Altogether, these results suggest that JI017 may be a potent candidate for the management of oxaliplatin-induced neuropathy as it decreases pain, spinal TRPV1, and astrocyte activation.

## 1. Introduction

Oxaliplatin has been widely used and recommended as a first-line treatment for metastatic colorectal cancer [1,2]. The clinical efficacy of oxaliplatin is better than that of previously used drugs, such as cisplatin [3]. Despite its anti-tumor efficacy, the dose-limiting toxicity of oxaliplatin limits its use [4,5]. According to a report, about 90% of oxaliplatin-treated patients suffered acute pain, which can be initiated only 24 h after a single injection of oxaliplatin [6]. Cold and mechanical allodynia are common occurring symptoms in animal models of oxaliplatin-induced neuropathic pain [7,8]. However, no optimal drug, without any side effects, has been developed, and the underlying mechanisms of this neuropathy still need to be elucidated [9].

Transient receptor potential vanilloid 1 (TRPV1) is a non-selective cation channel that responds to physical or chemical stimuli, such as acid, capsaicin, or heat [10,11]. In the spinal cord, it is mainly expressed in the superficial area of the dorsal horn [12,13]. In several types of experimental models of neuropathies, such as sciatic nerve injury (SNL), diabetes, and paclitaxel models, TRPV1 expression in the spinal dorsal horn is upregulated [14,15,16]. Treatment with a TRPV1 antagonist attenuates pain behavior in the SNL model [17]. In an animal model of painful diabetic neuropathy, the upregulated spinal *TRPV1* mRNA was downregulated with pain amelioration following oral administration of ginger extract and 6-shogaol [18]. Moreover, blockade of spinal TRPV1 expression in *Trpv1* knockout (KO) mice affects pain behavior, as well the activation of spinal astrocytes [14,17]. In pain conditions, the effect of spinal TRPV1 on spinal astrocytic activation needs to be elucidated.

Spinal astrocyte activation is a representative phenotype of various pain states, such as inflammatory pain, nerve injury-induced neuropathy, and chemotherapy-induced neuropathy [19,20,21,22]. Once astrocytes are activated, they enhance the transmission of pain by releasing gliotransmitters, such as pro-inflammatory cytokines [23]. These pro-inflammatory cytokines, such as interleukin (IL)-1β, IL-6, and tumor necrosis factor-α (TNF-α), stimulate spinal dorsal horn neurons and reinforce their spontaneous excitatory post-synaptic current frequency [24]. In an animal model of oxaliplatin-induced neuropathic pain, several reports have shown a positive causality between glial activation and neuropathic pain behavior [25,26,27]. In addition, spinal glial activation and oxaliplatin-induced neuropathic pain occurred simultaneously after a single injection of oxaliplatin [22]. Pain behaviors, in various animal models, ameliorated after suppression of the activation of spinal astrocytes [20,22,27]. According to our previous studies, several medicinal herbs attenuated oxaliplatin-induced neuropathic pain by suppressing the activation of spinal astrocytes [22,28,29]. Thus, targeting spinal astrocytes could be a potential strategy to modulate neuropathic pain.

Despite its severity, there is no clear way to address oxaliplatin-induced neuropathic pain. Drugs, such as duloxetine or venlafaxine, are recommended; however, their use is limited because of adverse effects, such as somnolence, vertigo, and nausea [30,31]. Medicinal herbs have been considered as an alternative and complementary therapy because of their safety and low incidence of adverse effects [32,33,34,35]. We have dedicated a few years to elucidate the analgesic potency of medicinal herbs on oxaliplatin-induced neuropathic pain [22,28,29,36]. JI017 is composed of three medicinal herbs, namely, *Aconitum carmichaelii*, *Angelica gigas*, and *Zingiber officinale*. A recent study has revealed that JI017 has anti-tumor effects on prostate cancer cell lines [37]. *Aconitum carmichaelii* has been reported to be potent against nerve injury-, oxaliplatin-, and paclitaxel-induced neuropathy [38,39,40]. *Angelica gigas* showed anti-nociceptive properties by increasing the latency against heat stimuli [41]. In our previous study, *Zingiber officinale* administration attenuated oxaliplatin-induced cold and mechanical allodynia [36]. Based on the additive effects of its components, we postulate that JI017, which has anti-tumor potency, is a potential analgesic agent.

In this study, we investigated whether JI017 attenuates oxaliplatin-induced neuropathic pain. Furthermore, we studied the underlying mechanism of the effect of spinal TRPV1 alteration on the activation of astrocytes at the spinal dorsal horn in an oxaliplatin mouse model.

## 2. Results

### 2.1. Oral Administration of JI017 Decreased Cold Allodynia, but Not Mechanical Allodynia, Induced by Single Oxaliplatin Injection

**Figure 1:**
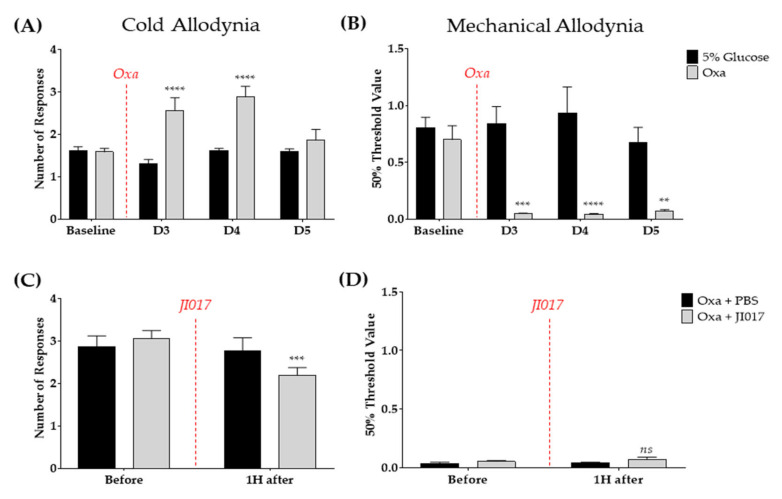
Oxaliplatin-induced cold and mechanical allodynia and the efficacy of JI017 on oxaliplatin-induced neuropathic pain. Changes in the behavioral response to acetone drop (**A**,**C**) and von Frey (**B**,**D**) stimulations were observed from day 3 (D3) to D5 after the oxaliplatin injection. On D4 after the oxaliplatin injection, when the cold and mechanical allodynia were significantly induced, JI017 or phosphate-buffered saline (PBS, control) were orally administered to the mice (**C**,**D**). The 5% glucose (intraperitoneal—i.p.) solution was used as a vehicle of oxaliplatin (6 mg/kg, i.p.) (**A**,**B**). PBS (per os—p.o.) solution was administered as control for JI017 (500 mg/kg, p.o.) (**C**,**D**). Baseline—before the administration of oxaliplatin or 5% glucose (**A**,**B**). Before—before JI017 or PBS treatment 4 days after oxaliplatin injection (**C**,**D**). Oxa—oxaliplatin. 5% glucose: *n* = 6, Oxa: *n* = 6, Oxa + PBS: *n* = 5, Oxa + JI017: *n* = 6. ns—non-significant, ** *p* < 0.01, *** *p* < 0.001, **** *p* < 0.0001 vs. 5% glucose or Oxa + PBS with two-way ANOVA followed by Sidak’s post-test for multiple comparisons.

In previous studies [36,42,43], a mouse model of cold and mechanical allodynia induced with a single injection of oxaliplatin was used to investigate the efficacy of JI017, a formula of medicinal herbs, and the mechanisms of its analgesic effect. First, the behavioral changes in response to cold and mechanical stimuli were observed from day 0 (D0) to D5. Similar to previous studies [36,43,44], oxaliplatin-induced cold and mechanical allodynia developed significantly from D3 to D5, in comparison with the control (vehicle) group, both peaking on D4 (Figure 1A,B). On D3 and D4, both cold and mechanical allodynia developed significantly (Figure 1A,B), and the mechanical allodynia lasted until D5 (Figure 1B). Second, JI017 (500 mg/kg) was orally administered on D4, and 1 h later, its anti-allodynic effect was evaluated. A preliminary study was conducted to determine the dose of JI017 (data not shown). Administration of JI017 suppressed cold allodynia, but not mechanical allodynia, in the oxaliplatin model (Figure 1C,D). Therefore, further experiments were conducted on D4, and the effects of JI017 were analyzed an hour after administration.

### 2.2. JI017 Down-Regulates Increased Spinal mRNA Level of TRPV1 and GFAP Following Oxaliplatin Treatment

**Figure 2:**
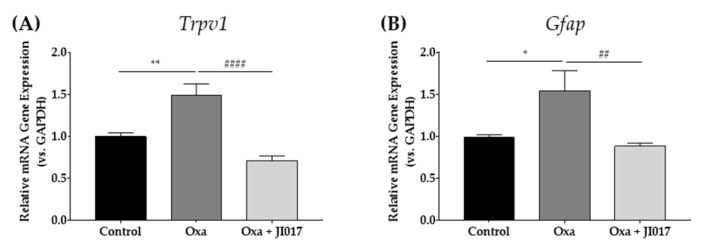
Changes in *Trpv1* and *Gfap* mRNA at the spinal level. On D4 after oxaliplatin injection, changes in *Trvp1* (**A**) and *Gfap* (**B**) mRNA levels were measured. In the oxaliplatin-treated group (Oxa), significant increase in spinal *Trpv1* and *Gfap* was observed compared with control. Upregulated spinal *Trpv1* and *Gfap* were recovered one hour after JI017 treatment (Oxa + JI017). Naïve mice were used as a control group (**A**,**B**). The Oxa group had administered PBS as a control for JI017. Control: *n* = 6, Oxa: *n* = 6, Oxa + JI017: *n* = 7. * *p* < 0.05, ** *p* < 0.01 vs. Control, ## *p* < 0.01, #### *p* < 0.0001 vs. Oxa with one-way ANOVA followed by Tukey’s post-test for multiple comparisons.

The effect of oxaliplatin treatment on the spinal mRNA levels of *the transient receptor potential cation channel subfamily V member 1 (Trpv1)* and astrocytes *glial fibrillary acidic protein* (*Gfap*) was investigated. According to several studies of neuropathic pain, including oxaliplatin-induced neuropathy, upregulated GFAP is manifested as the activation of astrocytes [20,27,45]. On D4, the mRNA levels of the spinal cords in the three groups were analyzed. In the oxaliplatin-treated group (Oxa), both *Trpv1* and *Gfap* were significantly upregulated compared with naïve mice (control vs. Oxa) (Figure 2A,B). In comparison with the oxaliplatin group, we tested whether JI017 modulated spinal *Trpv1* and *Gfap* changes after oxaliplatin injection. An hour after JI017 administration (500 mg/kg), both the upregulated *Trpv1* and *Gfap* expressions were significantly suppressed (Oxa vs. Oxa + JI017) (Figure 2A,B). JI017 treatment significantly downregulated *Trpv1* and *Gfap* expression in oxaliplatin-injected mice.

### 2.3. Increased Expression of TRPV1 and GFAP in the Superficial Laminae of the Spinal Dorsal Horn Was Attenuated after JI017 Treatment

**Figure 3:**
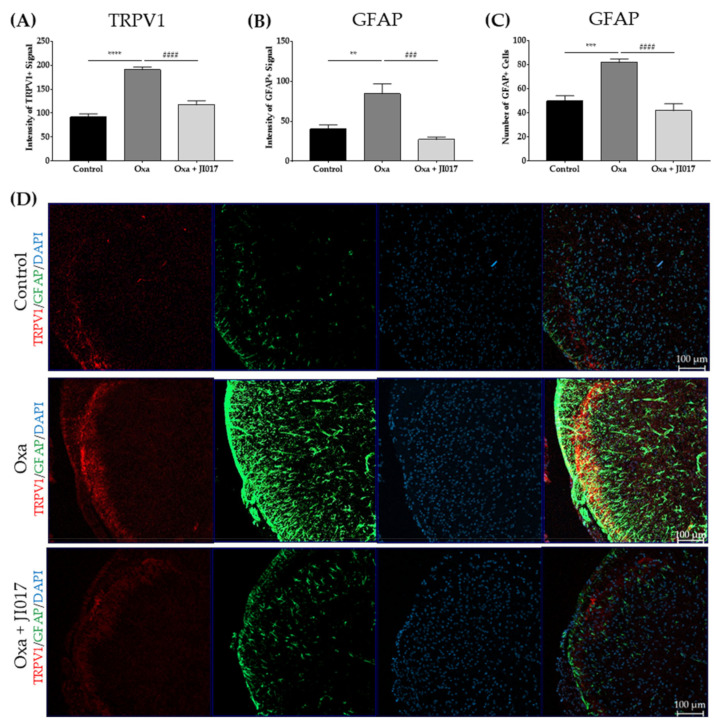
Changes of spinal TRPV1 and GFAP levels after JI017 treatment. On D4 after oxaliplatin injection, spinal TRPV1 and GFAP levels were observed and evaluated. In the oxaliplatin group (Oxa), an increase in spinal TRPV1 and GFAP signal intensity (**A**,**C**) and in the number of GFAP-positive cells (**B**) were observed. TRPV1 and GFAP signals were attenuated an hour after JI017 treatment (500 mg/kg). Naïve mice were used as controls (**A**,**B**). Mice of the Oxa group that were administered PBS were considered as the control of JI017. Representative images from each group were presented. The fourth image in each row represents the merge of the three images on the left (**D**). Control: *n* = 6, Oxa: *n* = 6, Oxa + JI017: *n* = 6. ** *p* < 0.01, *** *p* < 0.001, **** *p* < 0.0001 vs. control, ### *p* < 0.001, #### *p* < 0.0001 vs. Oxa, with one-way ANOVA followed by Tukey’s post-test for multiple comparisons.

Immunohistochemistry was used to determine whether oxaliplatin treatment could affect the TRPV1 and GFAP levels in the superficial laminae of the spinal dorsal horn. As mentioned above, spinal cords from all groups of mice were collected on D4. First, we noted that the intensity of the TRPV1-positive signal in the superficial area of the dorsal horn was strengthened (control vs. Oxa) (Figure 3A,D). Second, the intensity of the GFAP positive signal and the number of GFAP-positive cells was increased in the oxaliplatin-treated group (Figure 3B–D). To understand whether JI017 administration affects these two biomarkers upregulated by oxaliplatin, the levels of TRPV1 and GFAP in the superficial spinal dorsal horn were compared with those in the oxaliplatin group. JI017 was administered (500 mg/kg) to the oxaliplatin-treated group on D4, and spinal cords were collected 1 h after JI017 treatment. JI017 treatment significantly decreased the intensity of TRPV1- and GFAP-positive signals and the number of GFAP-positive cells in the dorsal horn (Figure 3A–D). We conclude that it effectively suppressed the expression of *TRPV1* and *GFAP* in the superficial dorsal horn.

### 2.4. Intrathecal TRPV1 Antagonist Injection Decreased TRPV1 and GFAP in the Spinal Dorsal Horn

**Figure 4:**
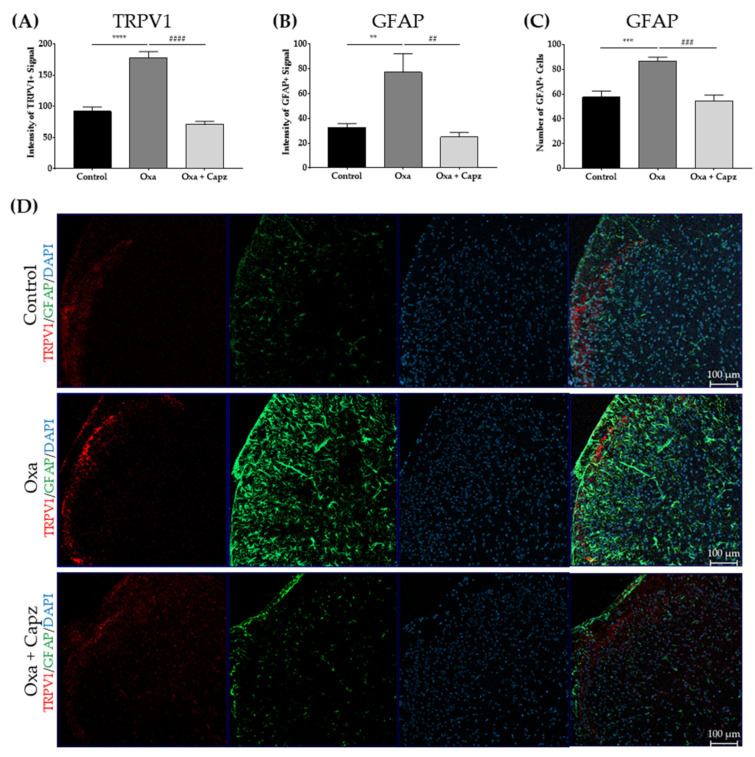
Effects of TRPV1 antagonist on spinal GFAP and TRPV1 levels. On D4 after oxaliplatin injection, spinal TRPV1 and GFAP levels were observed and evaluated. In the oxaliplatin group (Oxa), an increase in spinal TRPV1 and GFAP signal intensity (**A**,**B**) and in the number of GFAP positive cells (**C**) were observed. These increases in TRPV1 and GFAP signals were downregulated 2 h after capsazepine (Capz) treatment (10 μg). Naïve mice were used as a control group (**A**,**B**). Intrathecal injection of 20% DMSO was conducted on the Oxa group as a control for Capz. Representative images from each group are presented. The fourth image in each row represents the merge of the three images on the left (**D**). Control: *n* = 6, Oxa: *n* = 6, Oxa + Capz: *n* = 6. ** *p* < 0.01, *** *p* < 0.001, **** *p* < 0.0001 vs. control, ## *p* < 0.01, ### *p* < 0.001. #### *p* < 0.0001 vs. Oxa with one-way ANOVA followed by Tukey’s post-test for multiple comparisons.

To investigate the role of TRPV1 in spinal astrocytic activation, capsazepine, a TRPV1 antagonist, was intrathecally injected into oxaliplatin-treated mice. Two hours after the injection, the spinal cords were acquired. Immunohistochemistry was conducted to determine whether capsazepine treatment altered oxaliplatin-induced spinal astrocytic activation. On D4, an overexpression of spinal TRPV1 and GFAP in the superficial area was observed in the oxaliplatin group compared with naïve mice (Figure 3 and Figure 4). The intrathecal injection of capsazepine (10 μg) lowered TRPV1 levels in the superficial dorsal horn (Figure 4A,D). After 2 h of capsazepine treatment, the intensity of the GFAP-positive signal and the number of GFAP-positive cells in the superficial dorsal horn were significantly reduced (Figure 4B–D).

### 2.5. Oral Administration of JI017 Downregulated Increased c-Fos and TRPV1 in the Spinal Cord

**Figure 5:**
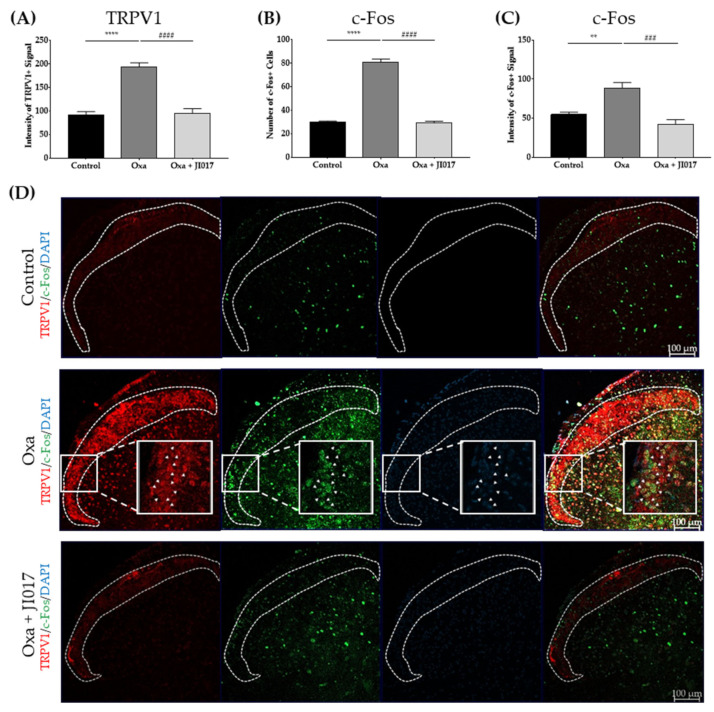
Oral JI017 administration altered the levels of c-Fos and TRPV1 in the spinal cord. Four days after oxaliplatin injection, the levels of spinal TRPV1 and c-Fos positive cells were evaluated (**A**,**C)**. In addition, c-Fos labeled neurons were also quantified (**B**). In the oxaliplatin group (Oxa), an increase in the intensity of the signal of spinal c-Fos and TRPV1 (**A**,**C**) and in the number of c-Fos positive cells (**B**) was observed. However, JI017 treatment (500 mg/kg) suppressed enhanced spinal TRPV1 and c-Fos intensity (**A**,**C**) and decreased the number of c-Fos positive cells (**B**). Each area encompassed by white dotted lines indicates laminae I and II (**D**). White arrowheads indicate co-localization of TRPV1 (red) and c-Fos (green) (**D**). Representative images from each group are presented (**D**). Control: *n* = 6, Oxa: *n* = 6, Oxa + JI017: *n* = 6. ** p < 0.01, **** p < 0.0001 vs. Control, *### p* < 0.001. *#### p* < 0.0001 vs. Oxa with one-way ANOVA followed by Tukey’s post-test for multiple comparisons.

To clarify whether JI017 could affect the activity of spinal TRPV1, levels of c-Fos and TRPV1 were evaluated in the L4/5 dorsal horn of treated mice. c-Fos expression in the spinal dorsal horn level has been used as an anatomical marker for neuronal activation related to pain [46]. Studies with neuropathic pain models reported a significant increase in the levels of Fos in the spinal dorsal horn [47,48,49]. In our study, 6 mg/kg of oxaliplatin-treated group showed an increase in c-Fos activity in the spinal dorsal horn on D4 (Figure 5B–D). However, oral JI017 administration (500 mg/kg) significantly suppressed the enhanced c-Fos activity in the spinal dorsal horn. Both the number of and the signal intensity in c-Fos-positive cells decreased (Figure 5B–D). Furthermore, as the co-localization of TRPV1 and c-Fos increased after oxaliplatin injection (Figure 5D, Oxa, white arrow heads), and decreased after JI017 treatment (Figure 5D, Oxa + JI017), JI017 may have inhibited the activity of TRPV1 channels and TRPV1 positive neurons in the spinal dorsal horn.

## 3. Discussion

Despite the prominent anti-cancer effect of oxaliplatin, severe neuropathy, induced even after a single injection, can lower the quality of life of patients and may lead to discontinuation of the treatment. Moreover, the drugs used to decrease this pain also have adverse effects, which limits their use [50,51,52]. In our study, the oral administration of JI017, a complex formula of medicinal herbs, attenuated oxaliplatin-induced neuropathic pain. While it significantly suppressed oxaliplatin-induced cold allodynia, it failed to attenuate mechanical allodynia. Additionally, JI017 administration led to a decrease in spinal TRPV1 and astrocytes, both of which had been upregulated after the oxaliplatin injection. Finally, an intrathecal injection of a TRPV1 antagonist, capsazepine, suppressed both the spinal TRPV1 expression and the activation of astrocytes, suggesting that JI017 may induce its anti-allodynic effect via spinal TRPV1 and astrocyte regulation. To our knowledge, this is the first study to analyze the activation of TRPV1 in oxaliplatin-treated mice.

Increased TRPV1 expression in the superficial laminae I–II of the spinal dorsal horn has been reported in various types of pain, such as complete Freund’s adjuvant (CFA)-induced inflammatory pain, sciatic nerve injury, diabetes, and paclitaxel-induced neuropathic pain [15,16,17,53]. In our study, too, the expression of spinal TRPV1 increased on D4 after oxaliplatin administration, when the pain behavior was the most developed (Figure 1, Figure 2 and Figure 3). This upregulation was counteracted by JI017 administration (Figure 2 and Figure 3). Furthermore, expression of c-Fos and TRPV1 significantly increased in the laminae I–II of the spinal dorsal horn after oxaliplatin injection and decreased following JI017 administration. Moreover, the increased intensity of c-Fos levelsin TRPV1-positive neurons decreased after JI017, suggesting that JI017 could inhibit the activity of TRPV1-positive neurons (Figure 5). However, calcium imaging or electrophysiology should be conducted to clearly understand the effect of JI017 on TRPV1 channels.

In our study, JI017 only attenuated cold allodynia, while mechanical allodynia remained unchanged. Although the involvement of TRPV1 in mechanical pain has also been reported in some studies [9,10], several studies have suggested that suppressing or ablating spinal TRPV1 alleviates thermal but not mechanical pain because only thermal hyperalgesia, but not mechanical allodynia, associated with both inflammation and nerve injury, was alleviated in TRPV1 knock-out mice [11,12]. Furthermore, TRPV1 antagonist treatment was not effective against mechanical sensitivity in CFA-induced pain [13]. In line with these results, intrathecal injection of resiniferatoxin, which impaired the spinal TRPV1, attenuated streptozotocin-induced thermal allodynia and decreased spinal TRPV1 expression [14]. In addition, oral administration of *Zingiber officinale* downregulated mRNA levels of spinal TRPV1 and suppressed thermal allodynia in the same diabetic model [15]. Moreover, intraperitoneally injected AMG9810, a TRPV1 antagonist, lowered thermal sensitivity in the partial sciatic nerve ligation (pSNL) model [16]. Moreover, lowered thermal sensitivity was observed in *TRPV1* knockout (KO) mice that had pSNL [16,17]. It is known that *TRPV1* is expressed mostly in C fibers, which are known to be cold-sensitive fibers [18,19]. This may explain, in part, the reason why only cold, but not mechanical allodynia was reduced after JI017 administration. Another explanation could be related to poor CNS penetration of JI017. In the study of Cui et al. [20], oral or intrathecal administration of either A-784168 (which has a good CNS penetration) or A-795614 (which has a poor CNS penetration), which are TRPV1 antagonists with similar in vitro potency, blocked capsaicin-induced thermal hyperalgesia with the same potency. However, in mechanical allodynia, the two compounds showed similar effect after intrathecal administration, whereas when administered orally, A-784168, which has a good CNS penetration, was much more potent than A-795614. They further stated that significant CNS penetration is necessary for a TRPV1 antagonist to produce an anti-allodynic effect on both thermal and mechanical pain. Based on this study, we speculated that orally administered JI017 may not have sufficiently penetrated the blood–brain barrier (BBB), which have resulted in failure of mechanical allodynia attenuation. To confirm this hypothesis, future experiments should be conducted by directly injecting JI017 into the spinal cord.

Along with spinal TRPV1, oxaliplatin also elicited increased activation of astrocytes in the spinal cord (Figure 2 and Figure 3). Several studies have related the suppression of spinal astrocytes activation to the analgesia of oxaliplatin-induced neuropathic pain [20,22,29,45]. Although the mechanism by which astrocytes are activated in pain conditions remains to be elucidated, a close relationship between spinal TRPV1 and astrocytes in pain development has been reported [14], as the activity of both increased in various animal models of pain [17,54,55]. In the CFA model, the administration of diacerein, which has anti-arthritic properties, lowered inflammatory pain behavior and alleviated the upregulation of spinal TRPV1 and astrocytes [53]. In the pSNL pain model mice, *TRPV1* KO lowered the activation of spinal astrocytes, and heat hypersensitivity was attenuated compared with the sham control [14,17]. The underlying mechanism by which spinal TRPV1 modulates the activation of astrocytes has not yet been clarified; while it is difficult to clarify based on our results, it may be possible that glutamate is involved in the TRPV1-mediated communication between neurons and glia [56]. Several studies have reported that TRPV1-expressing primary afferents are glutamatergic and that TRPV1 increases glutamate levels in the spinal dorsal horn [12,57]. In a study, SB-366791, a TRPV1 antagonist, inhibited glutamatergic transmission of C fibers in the spinal dorsal horn [58]. It should be noted that increased glutamate can sensitize glutamate receptors in astrocytes, leading to a Ca^2+^ influx and resulting in their activation [59,60]. Another chemotherapeutic agent, paclitaxel, also elicited TRPV1 expression and spinal astrocyte activation in primary afferent fibers originating from the dorsal root ganglion [16,61]. However, whether spinal astrocytes express *TRPV1* remains controversial. According to a study conducted on rats, 7% of the total TRPV1 is localized in spinal astrocytes [55]. Our study did not explore the co-localization of TRPV1 and GFAP signals in the spinal dorsal horn, and the possible involvement of astrocytic TRPV1 in neuropathic pain needs further study.

Medicinal herbs have been used to manage pain and treat various diseases since ancient times. Recently, they have attracted attention as alternative therapies because of their potency and safety [35]. JI017 consists of three medicinal herbs: *Aconitum carmichaelii*, *Angelica gigas*, and *Zingiber officinale*. In a previous study, JI017 showed anti-tumor potency in prostate cancer cell lines [37]. Although the analgesic efficacy of JI017 has never been reported, that of each of its components, in various pain models, has been documented [36,39,41]. We previously demonstrated the analgesic potency of *Zingiber officinale* in alleviating oxaliplatin-induced cold and mechanical allodynia in mice; 300 mg/kg of *Zingiber officinale* had higher efficacy than 100 mg/kg [36]. As *Zingiber officinale* only constitutes a quarter of JI017, in this study, it might have been in insufficient quantity to be efficacious against both cold and mechanical allodynia. Furthermore, HPLC data identified decursin, nodakenin, aconitine, and 6-gingerol as major phytochemicals of JI017 [37]. Decursin is a major phytochemical of *Angelica gigas*, while aconitine is present in *Aconitum carmichaelii*. 6-Gingerol is a key phytochemical of *Zingiber officinale*. Among the identified phytochemicals, it has been reported that decursin, aconitine, and 6-gingerol, can be delivered across the BBB [62,63,64]. Accumulating evidence suggests that phytochemicals can affect the central nervous system by crossing the BBB. Thus, these phytochemicals may have played a role in the analgesic effect of JI017 as decursin has been reported to attenuate paclitaxel-induced pain and to act as an TRPV1 antagonist [65], while aconitine has also been shown to have an analgesic effect on various animal models of pain [66]. The analgesic effect of 6-gingerol was also reported in several studies [67,68,69]. In addition, ligustilide and bulleyaconitine, major subcomponents of *Angelica gigas* and *Aconitum carmichaelii*, respectively, have shown potency in inflammatory and nerve injury-induced neuropathic pain by suppressing the activation of spinal astrocytes [70,71]. Moreover, 6-shogaol, a key phytochemical of *Zingiber officinale*, and ginger extract achieved pain relief by downregulating the mRNA of spinal TRPV1 [18]. However, whether phytochemicals from JI017 can suppress oxaliplatin-induced cold allodynia through the inhibition of spinal TRPV1 and astrocytes needs further study.

In conclusion, we demonstrated that a single oral administration of JI017 could suppress cold but not mechanical allodynia in mice. Spinal expression of both *Trpv1* and *Gfap* increased after single oxaliplatin treatment, whereas JI017 attenuated both. Moreover, our data showed that spinal TRPV1 inhibition by intrathecally injected TRPV1 antagonist blocked the activation of spinal astrocytes, suggesting that JI017 mediates its effect via spinal TRPV1 and astrocytes. However, more well-designed studies need to be conducted to clearly understand the relationship between TRPV1 and astrocytes in pain. Our study of JI017 would be a cornerstone for treating oxaliplatin-induced neuropathic pain by spinal TRPV1 and astrocyte regulation.

## 4. Materials and Methods

### 4.1. Animals

Adult C57BL/6 mice (6 weeks old) were obtained from DBL (Chungcheongbuk-do, South Korea) and housed in a specific pathogen-free (SPF) animal center. The animals were randomly distributed in cages. All these 96 mice were kept in a room with a temperature of 23 ± 2 °C, humidity of 65 ± 5%, fixed 12 h light/dark cycle, and with food and water ad libitum. All experimental protocols were approved by the Kyung Hee University Animal Care and Use Committee (KHUASP(SE)-20-448) on 15 November 2020 and were conducted in accordance with the guidelines of the International Association for the Study of Pain [72].

### 4.2. Preparation and Administration of Oxaliplatin

Oxaliplatin (Sigma Aldrich, St. Louis, MO, USA) was dissolved in a 5% glucose solution at a concentration of 2 mg/mL, as previously described [36]. A dose of 6 mg/kg was administered to the mice intraperitoneally. For the control, the same amount of 5% glucose solution was used.

### 4.3. Preparation and Administration of JI017

The JI017 (Hanpoong Pharm and Foods, Seoul, Korea) was obtained as a lyophilized powder. The dried roots of *Angelica gigas* and *Zingiber officinale* plants were purchased from GM PHARM (Gyeonggi, South Korea), and the dried root of *Aconitum carmichaelii* plant was purchased from Shin Hung PHARM. (Jeonnam, South Korea). The roots were boiled for 3 h in distilled 70% ethanol. The extract was filtered twice through Whatman grade 2 filter paper (GE Healthcare Life Sciences, Marlborough, MA, USA) to remove insoluble materials. The filtered extract was lyophilized to a powder using a freeze dryer (iLShin biobase, Gyeonggi, South Korea) and stored at 4 °C [37]. For administration, it was dissolved in phosphate-buffered saline (PBS), and the solution was administered orally at a dose of 500 mg/kg. All mice were administered with 300 μL of JI017 solution or PBS 4 days after oxaliplatin injection, when cold and mechanical allodynia were strongly induced.

### 4.4. Capsazepine Treatment

Capsazepine (Sigma Aldrich, St. Louis, MO, USA), a TRPV1 antagonist, was dissolved in 20% DMSO by vortexing. On D4 after oxaliplatin injection, capsazepine (10 μg) was intrathecally injected, and after 2 h, the mice were sacrificed.

### 4.5. Behavioral Assessments

Behavioral tests were conducted to assess the degree of allodynia in mice as described in our previous study [36]. All behavioral analyses were performed blindly. To assess whether oxaliplatin administration induced cold and mechanical allodynia in mice, behavioral tests were conducted before (baseline) administration of oxaliplatin or 5% glucose, and then at 3 (D3), 4 (D4), and 5 (D5) days after their injection. Cold allodynia and mechanical allodynia were measured using the acetone drop and von Frey filament tests, respectively. For acclimation, the animals were placed on a metal mesh floor and caged in an inverted clear plastic cage (12 × 8 × 6 cm^3^) for 30 min before all behavioral tests. To assess the behavioral responses to cold stimuli, an acetone drop (10 μL) was applied to the mid-plantar hind paw of the mice. Behavioral responses (flicking and licking) against the stimulus were observed and counted for 30 s. Thus, the term “number of responses” mentioned in the figures represents the average number of responses to an acetone drop in 30 s.

To measure mechanical allodynia, a series of von Frey filaments with bending forces of 0.02, 0.04, 0.07, 0.16, 0.4, 0.6, 1, 1.4, and 2 g (Stoelting, Kiel, WI, USA) was applied to the mid-plantar hind paws. The Dixon’s up-down method and Chaplan’s calculation method were used, and a withdrawal threshold of 15 g was applied as the cut-off [73,74]. The results obtained from both hind paws were averaged.

### 4.6. Grouping and Time Schedules of Behavior Test

To observe oxaliplatin-induced cold and mechanical allodynia, the animals were arbitrarily divided into two groups: oxaliplatin- (Oxa, *n* = 6) and vehicle- (5% glucose, *n* = 6) treated group (Figure 1A,B). On D4 after oxaliplatin injection, when the cold and mechanical allodynia were significantly induced, JI017 (Oxa + JI017, *n* = 6) or PBS (Oxa + PBS, *n* = 5) was orally administered. PBS was used as a control to JI017 (Figure 1C,D).

For spinal mRNA evaluation, animals were allocated into three groups: naïve mice (control), oxaliplatin-treated mice with PBS (Oxa), and JI017-on-oxaliplatin-treated mice (Oxa + JI017). Immunohistochemistry (IHC) was conducted three times. First, to observe the change after JI017 treatment, animals were grouped into three groups: naïve mice (control), oxaliplatin-treated mice with PBS (Oxa), and JI017-on-oxaliplatin-treated mice (Oxa + JI017). Second, to analyze the effect of capsazepine, mice were divided into three groups: naïve mice (control), oxaliplatin-treated mice with 20% DMSO (Oxa), and capsazepine- on oxaliplatin-treated mice (Oxa + Capz). Third, to investigate the changes of c-Fos activity after JI017 treatment, animals were grouped into three groups: naïve mice (control), oxaliplatin-treated mice with PBS (Oxa), and JI017-on-oxaliplatin-treated mice (Oxa + JI017). (Table 1).

### 4.7. RNA Extraction, cDNA Synthesis, and Real-Time PCR

Because oxaliplatin-induced cold and mechanical allodynia peaked on D4 and behavioral tests were conducted on D4, the animals were perfused with PBS, and the lumbar segments of the spinal cord were collected on D4. The collected spinal cords were homogenized using easy Blue (iNtRON Biotechnology, Seongnam, Korea) solution, and RNA was extracted from the spinal cord according to the manufacturer’s protocol. The extracted RNA was qualified and quantified by NanoDrop (ThermoFisher, Waltham, MA, USA) and converted to complementary DNA (cDNA) using a cDNA synthesis kit (iNtRON Biotechnology, Seongnam, Korea). The mRNA levels of TRPV1 (*Trpv1*) and GFAP (*Gfap*) were determined using SYBR Green qPCR Mastermix (Bioline Reagents, London, UK) in a CFX Connect Real-Time PCR system (Bio-Rad Laboratories, Hercules, CA, USA). Data were expressed as the ratio of targeted mRNA to glyceraldehyde 3-phosphate dehydrogenase (GAPDH) mRNA (relative mRNA expression). Table 2 lists the PCR primers used in the experiments.

### 4.8. Immunohistochemistry

The animals were deeply anesthetized with isoflurane inhalation (5% in 1:1 N_2_O:O_2_, *v*/*v*). They were perfused with 0.1 M PBS, 4% paraformaldehyde (PFA) (BBC Biochemical, Mt. Vernon, WA, USA) by cardiac puncture. The spinal lumbar segments L4/5 were exposed by laminectomy. By tracing the projected dorsal roots from their respective dorsal root ganglia, the L4/5 segments were identified. The collected tissues were post-fixed in 4% PFA overnight at 4 °C and then soaked in 30% sucrose (Sigma Aldrich, St. Louis, MO, USA), dissolved in 0.1 M PBS, for 48 h at 4 °C. Using the optimal cutting temperature (OCT) compound (Sakura Finetek, Tokyo, Japan), lumbar segment ice molds were prepared. These were sectioned into 20 μm thick slices using a cryostat (Microm HM 505N; Thermo Scientific, Waltham, MA, USA). The slices were mounted onto glass slides (Matsunami, Osaka, Japan) and dried for 3 h. These were then rinsed three times with PBS and then incubated for 45 min in 0.2% Triton X-100 in PBS at room temperature (RT). The blocking procedure was then performed using 3% bovine serum albumin (Bovogen Biologicals, Keilor East VIC, Australia) diluted in 0.05% phosphate buffered saline with Tween 20 (PBST) solution (30 min, RT). The glass slides were again rinsed with PBS and incubated overnight at 4 °C with primary antibodies: rabbit anti-GFAP (1:500; Cell Signaling Technology, Danvers, MA, USA) and mouse anti-TRPV1 (1:100; BD, Franklin Lakes, NJ, USA). For c-Fos staining, slides were incubated for 60 min in 5% bovine serum albumin in 0.3% Triton X-100 solution. After PBS rinsing, slides were incubated overnight at 4 °C with primary antibodies: rabbit anti-c-Fos (1:1000; Dako, Santa Clara, CA, USA) and mouse anti-TRPV1 (1:100; BD, USA). After rinsing again in PBS, secondary antibodies—anti-rabbit and anti-mouse immunoglobuling G (IgG) labeled with Alexa Fluor 488 and Alexa Fluor 594 (1:1000; Invitrogen, Waltham, MA, USA), respectively—were embedded at RT, in the dark, for 2 h. Immunofluorescent images were acquired using confocal laser microscope (LSM 800, Zeiss, Oberkochen, Germany) with a 10 × 0.45 numerical aperture (NA) objective lens. Quantitative analysis of GFAP- and TRPV1-positive cells in the spinal dorsal horn and co-localization of GFAP- and TRPV1-positive signals were performed using ImageJ software (National Institutes of Health, Bethesda, MD, USA).

### 4.9. Statistical Analysis

All data were presented as mean ± standard error of mean (SEM). Statistical analysis and graphic work were performed using Prism 7.0 (GraphPad software, La Jolla, CA, USA). One-way and two-way analysis of variance (ANOVA) followed by Sidak’s or Tukey’s post-hoc test for multiple comparisons were used for statistical analyses. In all cases, *p* < 0.05 was considered to indicate significant differences.

## Figures and Tables

**Table 1:** Experimental groups.

Pain Assessment (Figure 1)	mRNA Analysis (Figure 2)	IHC: TRPV1 and GFAP (Figure 3)	IHC: TRPV1 and GFAP (Figure 4)	IHC: TRPV1 and c-Fos (Figure 5)
5% Glucose (*n* = 6)	Control (*n* = 6)	Control (*n* = 6)	Control (*n* = 6)	Control (*n* = 6)
Oxa (*n* = 6)	Oxa (*n* = 6)	Oxa (*n* = 6)	Oxa (*n* = 6)	Oxa (*n* = 6)
Oxa + PBS (*n* = 5)	Oxa + JI107 (*n* = 7)	Oxa + JI107 (*n* = 6)	Oxa + Capz (*n* = 6)	Oxa + JI017 (*n* = 6)
Oxa + JI107 (*n* = 6)				

**Table 2:** Polymerase chain reaction (PCR) primers.

Gene	Forward	Reverse
*GAPDH*	5′-AGGTCGGTGTGAACGGATTTG	5′-GGGGTCGTTGATGGCAACA
*TRPV1*	5′-TCTCCACTGGTGTTGAGACG	5′-GGGTGTTTGAACTCGGTGTC
*GFAP*	5′-CGGAGACGCATCACCTCTG	5′-AGGGAGTGGAGGAGTCATTCG

## Data Availability

Not applicable.
